# Notes and News

**Published:** 1896-01-25

**Authors:** 


### NOTES AND NEWS.

(COLLECTED AND COMMUNICATED.)

A COMPETITIVE examination for seventeen commissions in the Army Medical Staff will be held on February 7th and following days. Particulars thereof will be found in our advertisement columns.

THE Royal Hospital, Richmond, has benefited substantially by its recent festival. It is actively and carefully administered, and the fact that the management only reaches 5 per cent. of the total expenses does the institution great credit.

SOME weeks ago mention was made of the great bazaar to be held at St. Petersburg for the benefit of a home for convalescents and invalids. This was formally opened just before the New Year, the Emperor and Empress performing the ceremony in the new rooms of the Hermitage, adjoining the Winter Palace, at St. Petersburg. The inauguration of this monster bazaar was of an imposing character, many of the members of the Imperial family being present, who further expressed their sympathy with the movement by making purchases at the stalls.

MUCH comment and indignation have been caused by the statements made by Mr. Murdoch, M.P., chairman of the Great Northern Central Hospital, with reference to the salaries paid to the secretaries of various hospitals. Surprise is expressed that, despite the protest of the secretaries, Mr. Murdoch, although challenged, has neither substantiated nor withdrawn the statements he made, an attitude which is untenable in the case of anyone holding a public position. We have reason to believe that several secretaries have requested Mr. Murdoch to do them justice by correcting his original statement so as to make it agree with the facts. Rumours have reached us that Mr. Murdoch contemplates retiring from the chairmanship of the Great Northern Central Hospital, but whether this be true or not these utterances of his at the special meeting of governors demand his prompt attention, for if they are much longer ignored they cannot fail to damage his reputation for impartiality and accuracy.

THE foundation-stone of a new eye, ear, and throat hospital has been laid by the Mayor at Cork.

THE Duke of Connaught will open the new wing of St. Thomas's Hospital on February 11th.

THE Hon. W. F. D. Smith, M.P., has consented to preside at a festival dinner in aid of the funds of the St. George's and St. James's Dispensary, 60, King Street, Regent Street, on Tuesday, February 18th.

THE sum collected for the Hospital Sunday Fund at Birmingham amounts to £4,422 this year. This sum shows a decrease of £116 on the previous year's collection. As far as the General Hospital is concerned the loss is more apparent, as the last time the collection was handed over to it, three years ago, the amount was greater by upwards of £600.

DR. SCHOTT's treatment for heart disease is now practised at Llangammarch, in Wales, which possesses a barium spring. Dr. John Williams referred on a recent occasion in favourable terms to Llangammarch Wells as a useful addition to the list of British health resorts. The district has been opened up through the enterprise of Mr. J. Griffiths, master of the Paviors' Company. There is now comfortable accommodation at Llangammarch for visitors, to whom the beautiful surroundings and invigorating air will be attractive.

IN a recent issue of our journal we drew attention to the value of the system of Pasteurising milk, and expressed a wish that the method might prove commercially successful, and thus capable of general adoption. This seems likely to be the case, for M. Contant, an engineer, has invented a very simple process of securing the desired sterilisation. The milk is placed in open bottles made of specially prepared glass, and raised to a temperature of 80 C. by the aid of steam. The bottles are sealed, and then cooled down suddenly. The operation lasts only a few minutes, and the milk is stated to be in good condition for 48 hours.

AT the last meeting of the delegates of the Metropolitan Hospital Saturday Fund, held under the presidency of Mr. Acland, the chairman of the council, it was unanimously resolved to distribute the sum of £17,650 among 174 institutions. This is £42 more than last year, but the participators have increased by 9. The institutions include 32 general hospitals, £6,608 10s.; 64 special hospitals, £6,101 2s.; 20 convalescent homes, £1,566 3s.; 36 dispensaries, £952 9s.; and 22 miscellaneous, including the Surgical Appliances Committee and the Society for Gratuitous Nursing of the Sick Poor in their Own Homes, £2,421 16s. The total receipts for the year amounted to £20,039, as against £19,978 last year. The principal recipients were the London Hospital, £820 2s.; Guy's, £535 13s.; St. Thomas's (first time of participation), £504 3s.; Brompton (consumption), £479 9s.; St. George's, £447 18s.; Middlesex, £391 3s.; St. Mary's, £375 11s.; City of London (chest), £342 11s.; University, £317 16s.; Seamen's, £309 9s.; Westminster, £297 3s.; Metropolitan Convalescent Home, £260 2s.; Morley House Convalescent Home, £237 12s.; National Hospital for Epilepsy, £231 2s.; Hospital for Sick Children, £217 7s.; Royal Free, £212 4s.; Royal London Ophthalmic, £200 8s.; Victoria Hospital for Children, £191 8s.; Surgical Appliances Committee, £1,000.

